# Analysis of drug resistance to HIV-1 protease using fitness function in genetic algorithm

**DOI:** 10.1186/1471-2334-12-S1-O7

**Published:** 2012-05-04

**Authors:** A Harishchander, S Senapati, D Alex Anand

**Affiliations:** 1 Department of Bioinformatics, Sathyabama University, Chennai, India; 2 Department of Biotechnology, Indian Institute of Technology Madras, Chennai, India

## Motivation

Analysing the potential organic molecule for inhibiting HIV-1 protease against its drug resistance by predicting its fitness using Genetic Algorithm will enhance research in the discovery of identifying the potential lead for inhibiting the aspartyl protease of HIV type I.

## Methods

Drug resistance is predicted for all FDA approved HIV-1 protease inhibitors and organic leads synthesized by Dr. Deeb and Dr. Godzari with wild type and mutant strains of subtype B. Initially the structural feature of HIV-1 protease with the inhibitor complex has been anlysed on the basis of “Binding Energies”. Finally the fitness function in Genetic Algorithm was used for optimizing the inhibition of specific organic lead with three fold cross validation.

## Results

Structural data mining performed by the fitness function in Genetic Algorithm gave pattern identities between HIV-1 protease (wild type and mutants) of sub type B against organic leads and FDA approved inhibitors of HIV-1 protease. Genetic Algorithm gives“80% Accuracy” for wild type inhibition and “75% Accuracy” for mutant inhibition in the final optimization by fitness function.

## Conclusion

Organic leads have greater affinity than the FDA approved inhibitors (specifically Mol-23 which has good correlation with pIC50 and H Bonding descriptors). I84V mutant still remains resistant to both FDA approved Inhibitors and Organic Molecules. In future the dynamics of the molecule will be analysed for all FDA approved protease Inhibitors and potential organic leads with the wild type and mutant proteases of HIV type I.

